# How Can Research Keep Up With eHealth? Ten Strategies for Increasing the Timeliness and Usefulness of eHealth Research

**DOI:** 10.2196/jmir.2925

**Published:** 2014-02-19

**Authors:** Timothy B Baker, David H Gustafson, Dhavan Shah

**Affiliations:** ^1^Center for Tobacco Research and InterventionSchool of Medicine and Public HealthUniversity of Wisconsin - MadisonMadison, WIUnited States; ^2^Center for Health Enhancement Systems Studies (CHESS)Industrial & Systems EngineeringUniversity of Wisconsin - MadisonMadison, WIUnited States; ^3^Mass Communication Research CenterSchool of Journalism and Mass CommunicationUniversity of Wisconsin - MadisonMadison, WIUnited States

**Keywords:** social media, Internet, randomized clinical trials, experimental designs, research techniques, patient education, patient engagement, health communication, telemedicine

## Abstract

**Background:**

eHealth interventions appear and change so quickly that they challenge the way we conduct research. By the time a randomized trial of a new intervention is published, technological improvements and clinical discoveries may make the intervention dated and unappealing. This and the spate of health-related apps and websites may lead consumers, patients, and caregivers to use interventions that lack evidence of efficacy.

**Objective:**

This paper aims to offer strategies for increasing the speed and usefulness of eHealth research.

**Methods:**

The paper describes two types of strategies based on the authors’ own research and the research literature: those that improve the efficiency of eHealth research, and those that improve its quality.

**Results:**

Efficiency strategies include: (1) think small: conduct small studies that can target discrete but significant questions and thereby speed knowledge acquisition; (2) use efficient designs: use such methods as fractional-factorial and quasi-experimental designs and surrogate endpoints, and experimentally modify and evaluate interventions and delivery systems already in use; (3) study universals: focus on timeless behavioral, psychological, and cognitive principles and systems; (4) anticipate the next big thing: listen to voices outside normal practice and connect different perspectives for new insights; (5) improve information delivery systems: researchers should apply their communications expertise to enhance inter-researcher communication, which could synergistically accelerate progress and capitalize upon the availability of “big data”; and (6) develop models, including mediators and moderators: valid models are remarkably generative, and tests of moderation and mediation should elucidate boundary conditions of effects and treatment mechanisms. Quality strategies include: (1) continuous quality improvement: researchers need to borrow engineering practices such as the continuous enhancement of interventions to incorporate clinical and technological progress; (2) help consumers identify quality: consumers, clinicians, and others all need to easily identify quality, suggesting the need to efficiently and publicly index intervention quality; (3) reduce the costs of care: concern with health care costs can drive intervention adoption and use and lead to novel intervention effects (eg, reduced falls in the elderly); and (4) deeply understand users: a rigorous evaluation of the consumer’s needs is a key starting point for intervention development.

**Conclusions:**

The challenges of distinguishing and distributing scientifically validated interventions are formidable. The strategies described are meant to spur discussion and further thinking, which are important, given the potential of eHealth interventions to help patients and families.

## Introduction

eHealth interventions are appearing and changing so quickly that they are challenging the way we conduct health care research. For the purposes of this paper, we adopt a broad definition of eHealth in keeping with Eysenbach’s definition [1]. That is, we view eHealth as an attempt to enhance health or health service delivery through use of modern information technology and electronic communication resources [2]. Thus, in our view, eHealth comprises interventions involving the Internet, wireless communications, interactive TV, voice response systems, kiosks, personal digital assistants (PDAs), CD-ROMs, DVD-ROMs, and remote monitoring that guides intervention delivery. Because eHealth interventions—for example, mHealth, telemedicine, information and communication systems—are defined in part by the technology they are built upon, their nature, relevance, appeal, and uniqueness are all affected by the rapid pace of technological change. By the time a randomized trial of a new intervention takes place, updated technology is likely to make the tested intervention and the results of the trial out-of-date. A recent paper [3] addressing the need to speed up the research enterprise noted that it often takes 7 years to submit a grant, design and pilot test the methods, conduct the research, analyze the data, and publish the results. If this span consisted of the years 2006-2012, the following innovations would have occurred in that period: the Wii, the iPhone, the Android system and products, the iPad, and Twitter. What have we learned about conducting research in such a fast-changing world and what can we do about it? The past gives us some guidance.

## A Brief History

We have conducted randomized trials on eHealth interventions since the 1970s for a variety of chronic conditions, such as cancer, asthma, and addiction [4-8]. When we started exploring the effectiveness of eHealth interventions [5,6,9,10], iPads, iPods, smartphones, Twitter, Facebook, and even the Internet were things of the future. We began by creating interventions to be used on desktop computers.

When we started, we believed desktop computers and material displayed on monitors would be an important way to communicate for decades. This assumption guided our work on the services contained in two early systems we developed, BARN [10] (the Body Awareness Resource Network, intended for adolescents) and CHESS (Comprehensive Health Enhancement Support System) [7]. For example, the early versions of our CHESS breast cancer interventions contained services such as an “Instant Library”, answers to “Frequently Asked Questions”, “Personal Stories” of patients struggling with breast cancer, a treatment decision support system, a discussion group, and “Ask the Expert”. Individually, these services were quite new and because they were integrated and coordinated within an invitation-only website (a “walled garden”), they were unique. Our randomized trials found that participants in our studies who had access to CHESS used it heavily and did better clinically than participants receiving usual care or who had unfettered access to the whole Internet but did not have access to CHESS [8,11,12]. In particular, we found that participants heavily used the “social” components of the system (ie, the discussion groups).

When we first started to test CHESS systems in the mid-1980s, we loaned participants desktop computers (Apple IIs) and arranged and paid for their dial-up connection (to either the Internet or to a computer-linked modem in pre-Internet days). This was the first time many participants had used a computer or the Internet. This no doubt motivated some participants to volunteer for the research and be active users of the new system [13-16].

Our early desktop/laptop-based interventions are now dated. We have trouble recruiting breast cancer patients into studies using desktop/laptop interventions, and once we recruit individuals into the research, participants do not use CHESS as much as they once did, especially the social resources. Moreover, desktop/laptop CHESS systems for breast cancer do not appear to confer the same benefits they once did [17].

PC-based CHESS systems for breast cancer were novel 20 years ago, but they now offer less functionality as a social resource than do websites such as Facebook. Some people may still prefer the “walled garden” of CHESS, which confers confidentiality and vets participants, but the ability to create “circles” within existing social networking platforms has lessened this distinction. In essence, about 10 years ago participants in our research found CHESS to be novel and appealing, and our research showed that it was heavily used and exerted large effects relative to access to other Internet resources. That has all changed substantially.

The speed of this transition is breathtaking and can be appreciated by comparing the shelf-life of CHESS with that of other sorts of psychosocial interventions: for example, with behavioral interventions that have existed in similar forms for the past 50 years and with psychodynamic psychotherapy, some forms of which are still being offered and used much as they were a century ago. Thus, in the past, researchers could afford to conduct research at a desultory pace; the results of their research would be relevant for many years. We believe that the history of CHESS is a harbinger of what is in store for virtually all eHealth intervention strategies. They will become dated and remarkably quickly. This will occur because a defining feature is the nature of their delivery mechanism(s)—which is vulnerable to the breakneck pace of technological change.

## The Challenges for Research

eHealth interventions take time to evaluate in part because they are so complex. They involve a combination of content, user, social interaction, platform, links, and interface, making them intrinsically complicated to study. An intervention might have greater real-world utility to the extent that it permits adaptation in these dimensions, yet this same complexity and adaptability can complicate the evaluation of an intervention (and of course, too much complexity might hinder use and efficacy). Is a cancer intervention the same if delivered on highly divergent platforms with unique functionality (smartphone vs desktop)? What is being evaluated if the intervention also permits broad access to the Internet? Is it the intervention or Twitter, Facebook, WebMD, or the Cancer Survivor Network that is driving the observed effect? Such adaptability may obfuscate the nature of the intervention [18], produce great variation or error in the effects of the intervention, and, because these linked resources will themselves change over time, the evaluation itself will be built on shifting sand.

The dissemination of eHealth resources (or related applications such as social media) can occur very rapidly. Thus, a commercial developer can produce a resource and release it in short order so that the resource is in the hands of thousands of users, with nothing being known about its efficacy. Such interventions may become more widely used than validated interventions for reasons other than their effectiveness (eg, search engine status or appearance). In few fields is an intervention made available to so many, so quickly, as in eHealth, with some even going viral. Rapid dissemination over the Internet not only increases the rate at which new interventions enter the field but also the rate at which extant interventions are rendered out-of-date and unappealing. Hence another irony: An intervention that is tested and experimentally validated before dissemination may become less widely used because its content, functions, and platform are no longer innovative by the time it is disseminated. The corollary is, of course, that the widely used, novel, and untested intervention may be inert or even harmful and could displace the use of effective interventions.

Even in the best of circumstances, when an intervention has been shown to be effective and intensively used, it will be copied by numerous competitors so that the research version of the intervention is ubiquitous, seems commonplace, and cannot be cleanly evaluated because individuals in control conditions have ready access to its components through competing systems. It would be hard to evaluate psychoanalysis if every friend or neighbor processed transference, did dream interpretation, and maintained therapeutic neutrality.

A final challenge to conducting research on eHealth interventions is the pace of *clinical* discoveries. When we first produced the CHESS breast cancer intervention, we made wholesale updates of the content annually. This periodicity was fine in the 1980s and 1990s, but faster updating must be done now as a consequence of accelerating progress in fields such as the radiology, oncology, and pharmacogenomics [19]. Efficient strategies can be used to maintain some currency, such as including links to current literature that can be updated quickly and cheaply. However, the core of the information presented by the intervention and its integration with such features as treatment decision-making need to be current or the intervention loses value and credibility among clinicians and patients. Finding and hiring highly skilled experts to help with the updating is difficult and expensive, and even a regularly updated eHealth intervention may have a short life. Again, we can evaluate a given instantiation of the intervention (including on-going attempts to keep it current), but its overall novelty and appeal can erode over the course of a study that may last years and will certainly change in dissemination.

## Increasing the Efficiency of eHealth Research

The rapid changes occurring in technology and the pace of medical research highlight a need for eHealth research strategies that both increase the pace of research and also produce higher-value interventions that will be more effective than the ascientific application of technology (eg, by app developers) and therefore, remain appealing and effective despite tumultuous change. In other words, researchers can improve eHealth by enhancing both the efficiency and rapidity of eHealth research and its quality and merit. We will start by suggesting ways to accelerate the pace of eHealth research and then consider how to enhance the quality of interventions.

### Think Small: Towards More Focused and Efficient Research Studies

Large clinical trials designed to evaluate the effects of whole eHealth interventions may take many years, considering the time needed to secure funding, conduct recruitment and implementation, and so on [3]. However, researchers can conduct small-N studies that efficiently target relatively discrete questions. This research can occur either in the laboratory setting (eg, if special equipment is needed such as eye-tracking or physiological apparati) or with small samples of real-world users (eg, if opportunities exist for examining existing systems). Many questions might be addressed effectively with small sample ad hoc experiments, which may allow many questions to be addressed simultaneously. Which tailoring features do people prefer? Which methods of framing information help individuals remember key points? The key is to detach addressing such questions from the analysis of an entire eHealth intervention that occurs in the context of a large and slow-to-complete clinical trial [20].

Certain strategies go hand-in-hand with this smaller, focused approach, beginning with using more proximal outcome measures, ones that are clinically meaningful but also highly sensitive and quickly responsive to the effects being evaluated [21]. One reason that AIDS research may have progressed rapidly compared with cancer research is its focus on “surrogate endpoints”, such as viral load, that are highly sensitive to therapeutic change and that have reduced reliance on distal clinical outcomes, such as survival duration [22]. This sort of focus might lead eHealth researchers toward using meaningful yet efficient outcomes such as self-efficacy, increased medication adherence, and greater perceived social support. Such outcomes could not substitute for vital clinical endpoints, but they could be used in ongoing research that results in continuous improvement [23].

Second, some experimental designs are geared for small sample studies. These designs are referred to with various labels such as “single-subject”, “multiple baseline”, “stepped wedge”, and “quasi-experimental” designs. These involve the systematic presentation and/or removal of interventions or intervention components from participants and determining whether meaningful change occurs contingent upon such manipulations [24,25]. Importantly, new analytic methods are appearing that increase the internal validity of such efficient strategies (eg, de Vries & Morey using Bayes’ tests for single-subject data [26]).

### Use Efficient Research Designs

Evaluation of eHealth treatments often occurs via randomized clinical trials. While there is a vital role for such trials, they often do not provide as much information as alternative experimental strategies. For instance, engineering researchers [23] typically use highly efficient factorial and fractional-factorial designs that allow for the testing of multiple hypotheses or interventions with no loss of power even as the number of tested interventions increases. Collins et al note that testing 6 intervention features or components would require 6 different studies if traditional randomized controlled trial (RCT) designs were employed (comparing an active component with a control/placebo component in each study) [23]. However, a single experiment could contrast all 6 intervention components if they were tested in a 6-factor factorial design (with each factor comprising an active and control component) with every participant being independently randomly assigned to each factor.

Factorial designs have some decided advantages over the traditional RCT approach. For example, the 6-factor factorial design is far more efficient; for example, using the same targeted effect size, the factorial experiment would have the same power to test each factor as would each RCT and use about one-sixth the participants. In addition, the factorial experiments would allow the investigator to estimate interaction effects among intervention components, which would indicate which combinations of intervention components worked best together—something not possible (efficiently) with conventional RCTs. And certainly the investigator could conduct the factorial experiment in less time than it would take to do 6 RCTs.

Other efficient research strategies or designs might also speed the research process, namely sequential, multiple assignment, randomized trials (SMART) or adaptive designs [27-29]. Such designs are appropriate for conditions or problems where a change in a patient’s status might require a change in treatment approach; for example, smoking treatment might be changed when a patient trying to quit relapses back to regular smoking. In SMART designs, the researcher may not only investigate multiple intervention components in the same study, but do so as participants transition across the various phases of recovery [20]. Further, one can also ask research questions that rely not on new interventions, but on systems already in use. This could reduce development time and costs and speed the dissemination of findings. One of the authors has recently modified the existing National Cancer Institute smoke-free website by recruiting smokers who visit the site and randomizing them to different website versions. With such a strategy, researchers can simultaneously evaluate reach, effectiveness, and maintenance [30] (see Riley for additional strategies to speed the research enterprise [3]). This strategy of using existing interventions and delivery systems seems especially appropriate when ambitious, comprehensive research questions are examined—for instance, those involving multiple aspects of effectiveness (eg, across communication, control, care, and contextual dimensions [20]). In this way, the time needed for development and implementation are not added to the time needed for evaluation.

### Study Universals

Research evaluating eHealth interventions often addresses the effects of a particular intervention, which can delimit the relevance of the research (eg, as the intervention becomes dated, so do the results of the research). To increase the odds that research yields durable and broadly relevant results, research could examine timeless behavioral, psychological, and cognitive principles and systems. These principles and systems are typically generated by well- supported basic theory (eg, theories of behavior change or quality of life, such as self-determination theory or other broad social science theories; see Kaplan et al [20] and Niemiec et al [31]). It is also possible, though, that theory that is more applied in nature could also reveal universal principles (eg, theories of general system design and others, such as Dansky [32] and Yen & Bakken [33]). What are the principles by which information is made more salient? How should people be queried to help them arrive at optimal decisions? What general approaches increase motivation in an eHealth context? What sorts of messages most efficiently provide emotional and instrumental support? Just as psychometricians validate an assessment instrument across multiple populations to reduce sampling error, eHealth researchers should validate principles across diverse interventions and platforms (eg, with efficient factorial designs), thus building into research the demonstration of broad relevance. Of course, such research should search for moderation effects to determine just how “universal” the phenomena or principles are. For instance, it may be the case that some technology-intensive interventions will be inappropriate for developing countries [34].

### Anticipate the Next Big Thing

Researchers need to anticipate eHealth strategies that will work in future environments, not in the present [35,36]. For instance, to figure out new approaches to what we regarded as an unsustainable addiction treatment system, we at the University of Wisconsin Center for Health Enhancement Systems Studies convened a meeting of drug addicts, family members, biomedical engineers, nano-technologists, futurists, computer scientists, and experts in social networking, quality improvement, and pharmacology. Only two people were from addiction treatment. The addicts and families told their stories. A futurist reviewed where the world was likely to go generally. The group was told that a virus had selectively killed every addiction treatment provider. The attendees had to design an addiction treatment system built solely on technology. Attendees broke into groups, each containing an addict, a family member, and experts. Many new insights emerged, including those that led to our work in smartphones and sensors [37]. The key to our innovation in this area was assuming that we could depart entirely from prior approaches to addiction treatment and to enlist very new outside perspectives in conceptualizing change.

The “next big thing” also certainly involves incorporating the latest technology into eHealth interventions. Using mobile devices has enabled us to create services we could not have imagined 20 years ago. We can build into CHESS features that take advantage of standard functions of smartphones, such as accelerometers, GPS, two-way video cameras, and magnetometers. These features allow us to create what are being called “ecological momentary assessments and interventions”. For example, the smartphone-based A-CHESS (Addiction—Comprehensive Health Enhancement Support system) includes a service to track the movement of people recovering from alcoholism. If a user gets near a bar he or she used to frequent, the GPS initiates a rescue service by sending alerts to the participant and making calls to pre-designated friends or family [38]. Some participants find it easier to listen to content than read it; on the smartphone, most content can be presented auditorily. Other sensors enable us to measure almost innumerable mental and physical capabilities, creating the opportunity to use the phone’s features to identify physical and mental indicators of stress and dysfunction and automatically request help. Using the latest technology and listening to new viewpoints in designing the A-CHESS intervention seem to be paying off; early results of this intervention have been very encouraging [39].

### Improve Information Delivery Systems

Even when researchers make important and timely discoveries, it is difficult and time-consuming to disseminate them to other researchers. Traditional dissemination vehicles such as conferences do not keep pace with rapid developments in research. A mantra of our research center is that no one should suffer twice. Dealing with an injury or disease is tough enough. Doing so in an inefficient, complex delivery system adds suffering. So, certainly attention to methods for improving communication and “handoffs” among providers, patients, and family should be a key goal of eHealth research [40-43]. Handoffs of results between researchers should similarly be improved by new, innovative methods—methods that apply the same conceptual analysis, effort, and information technology resources that are used in the designs of their eHealth interventions. Specifically, more research and effort need to be expended on vehicles and resources that allow eHealth researchers to communicate efficiently with one another and share research experiences, products, and resources (eg, prepublication findings, solutions to technical problems, coding resources, and so on). Such resources could support the development of research teams that share intervention development burdens and jointly recruit for studies and could foster the use of common measures that would promote “big data” research (see the section below, “Develop models, including mediators and moderators”). These steps could significantly accelerate the conduct of individual studies and the overall pace of research as well. Barriers to such developments certainly exist, such as concerns about authorship credit for developing interventions and reporting findings and the work and money needed to maintain such resources. But these barriers could be overcome (eg, by allowing website posting to constitute a claim to authorship). These sorts of resources would not compete with normal channels of research communication (eg, peer-reviewed journals) but would complement the research that appears in such outlets. This would require expansion of the sort of Web resources that have been developed for other research domains (eg, the research methodology website sponsored by Pennsylvania State University [44]). Certainly the communication and technological sophistication of eHealth researchers could be leveraged to address their own communication problems.

### Develop Models, Including Mediators and Moderators

While vast amounts of data exist from eHealth experiments, the data have not been mined systematically. eHealth has generated extensive databases. Enormous amounts of information lie within keystrokes and in messages, posts, and chats and can—with users’ permission—be analyzed to develop decision-support systems that help users address their concerns more efficiently and effectively. Mathematical modeling and simulation can help transform data into information. Bayesian models have been developed to predict whether a person will make an attempt on his or her life [5]. Simulations can rapidly compare treatment alternatives. Where data do not exist, methods for quantifying expert judgments can be employed [45]. The availability of such databases permits the evaluation of important research questions without developing interventions and implementing them or conducting new clinical trials. Such “big data” approaches to research would echo development in other areas of research such as dBGap, a repository for GWAS (genome-wide association study data) and related phenotype data. The researcher communication Web resources described above could similarly house eHealth datasets.

Large existing databases would be an ideal resource for conducting tests of mediation and moderation. Mediation analyses suggest how interventions work, and moderation analyses identify factors that modulate how well interventions work. Moderation analyses, when done with pre-existing datasets, would dramatically reduce the time spent in obtaining “generalizability data” [46], allowing researchers to discover facets across which findings can be generalized: In which persons and contexts does an intervention work well? These questions lie at the heart of most comprehensive eHealth evaluation models [20,32,33]. Researchers should also conduct mediational analyses across different contexts to find general mechanisms of effect [47]. Mediation research is important because it can tell us if our theory of the intervention is correct. Is it working as hypothesized? Discovering how right, or wrong, our theories are could save untold time that might be spent going down blind alleys. Moreover, mediation analysis can tell us not only what an intervention is doing, but also what it is *not* doing (eg, not increasing knowledge of treatment side effects, not improving affect). Such information is vital to efficiently revising both our theories and our interventions, and it can now be more efficiently implemented with the development of multiple-mediator analytic strategies [48].

## Improving the Quality of eHealth Interventions

### Continuous Quality Improvement

Possibly because researchers know that countless studies can be done to assess the effects of a particular intervention, they are reluctant to change an intervention before it has been thoroughly evaluated. This, of course, produces a stagnant island in a sea of change. The recent biography of Steve Jobs [49] relates that Jobs and his colleagues at Apple were often not the first to think of or develop a product. Various portable music players preceded the iPod, tablets pre-existed the iPad, and so on. But Apple made the product better than anyone else—for instance, easier to use and more elegant. Sometimes the product did not start out better, but became better through rapid quality improvement (the original iPad did not have a camera). Perhaps eHealth researchers need to think more like engineers, who tend to use the continuous improvement principle whereby every product is in a sense a beta version, that is, always the target of improvement and refinement, as in Collins [23]. This meshes with recent calls to conduct ongoing, systematic assessment of eHealth interventions across their life cycles [2,33]—assessment that taps diverse intervention dimensions and effects (eg, quality of use, impact on workflow, costs). While called for, such comprehensive iterative evaluation certainly remains rare [33].

If an eHealth intervention is repeatedly altered for purposes of quality improvement, how can its clinical effects be evaluated? Can one evaluate an intervention that never “stays still”? Actually, this is possible. One strategy would be to compare the intervention longitudinally through its various improvements against some reasonable control condition such as “adlib” Internet access, which itself would be changing over time. Advances in intensive longitudinal modeling [50] permit powerful and focused tests in such multiphased longitudinal datasets.

### Help Consumers Identify Quality

New eHealth developments are appearing rapidly, but “consumers” have very few ways to identify valuable ones (and consumers could include health care systems, clinicians, and others, in addition to patients and patients’ support networks). Many apps and eHealth interventions appear to have substantial weaknesses [51]. To complicate matters, eHealth systems are ever changing. Hence a one-time evaluation of an intervention can have limited value. Even identifying what new products exist can be very difficult. Consumers, like researchers, are challenged to keep up with such rapid development. New ways are needed to help consumers (and funders) make informed decisions about products. However, multiple barriers exist to addressing this need. For instance, rating eHealth quality comprehensively can be complex and difficult [51], especially if evaluations target product effectiveness or multiple intervention dimensions [52]. A more feasible approach might be to start by evaluating eHealth resources systematically with easy-to-assess criteria, for example, “transparency criteria” [51], such as disclosure of authorship, sponsorship, and/or ownership; recency of last update; authors’ credentials; nature of the review process for information accuracy; and so on. Other fairly brief rating systems such as DISCERN [53] might also be modified to provide relevant rating dimensions [54]. However, this still leaves unanswered questions such as who would conduct such ratings and how the ratings would be promulgated. These challenges may not be too daunting. For instance, a consortium of research organizations with some modest government sponsorship should be able to conduct ratings of numerous websites efficiently and cost-effectively. Assuming the use of an easy-to-use rating instrument, such ratings would be far less arduous and costly than Cochrane evaluations. Moreover, the promulgation and advertisements of such ratings would seem straightforward—all rated and approved websites could prominently display evidence of their meeting the quality rating criteria, and their communications could educate the public to turn to eHealth resources that meet and display such approval. In other words, the dissemination medium would be the rated websites themselves. It is important to note that once a mechanism for the relatively basic evaluation of eHealth resources is developed, this system could be used to support more ambitious evaluations of quality, such as those addressing evidence of accuracy, completeness, reading level, design, and effectiveness [51].

### Reduce Costs of Care

Reducing costs is often thought to be the enemy of quality, yet from numerous perspectives (societal, health care system), demonstrating cost reduction is very important and perhaps the chief means of increasing the dissemination of eHealth interventions (because it will appeal to decision-makers and purchasers). Yet, cost effectiveness or benefit is too infrequently demonstrated or identified as a key goal of research. For instance, reviews of available studies either report very little evidence on cost savings or specifically cite this as a lack in the field [55-58]. The impact of eHealth on costs will drive health system decisions for years to come. Properly designed, eHealth programs hold promise for reducing costs by speeding recovery and reducing admissions. eHealth might reduce the costs of care in many ways. For instance, informational resources might (1) reduce the frequency of medical staff contacts, (2) facilitate communication in chronic care intervention teams so that care is efficiently shifted to lower-cost providers, (3) directly deliver psychosocial interventions, thereby reducing the use of professional care, (4) improve patient preparation for health care visits, making them more efficient and reducing the need for repeat visits, and (5) improve patient satisfaction with care (eg, by increasing perceived connection with caregivers) and thereby reduce health care plan churn. Future research on eHealth interventions should explicitly consider the cost-effectiveness and cost-benefit impacts of an intervention (including formulating a business plan [59]), and, when possible, incorporate measures to address these outcomes.

### Deeply Understand Users

The rapid increase in new technologies raises the potential of innovation bias [60]—that is, developers becoming so infatuated with an innovation that user needs become secondary. Researchers and developers should deeply understand user assets and needs and how technologies can build on strengths to meet user needs. And it is important to recognize that users of the technology could be considered not only to be the people actually interacting with a device, such as patients and clinicians [61], but also closely linked others who are significantly affected by the device (eg, family members trying to provide care). When we began our University of Wisconsin Center of Excellence in Active Aging, the technical team (including programmers) visited the homes of frail elderly individuals, ate with elders at congregate eating facilities, and volunteered 4 hours a week at a senior center. This helped transform a job into a calling. It also helped reveal needs and assets that elders themselves did not recognize, which suggested innovative solutions. In one home, we observed an elderly person try to move without a walker and almost falling as a result. This taught us the danger for some of unaided movements of even a short distance. In response, we constructed the “screaming walker”, which has a radio-frequency identification chip that alerts the person when he or she tries to step away unaided. Because the in-depth assessment of user needs and assets is time consuming, developers often limit investment in this crucial activity. Fortunately, evidence shows that eHealth developers are increasingly making user input and needs assessment key elements in intervention design and practices that continue across intervention development [61].

## Discussion

### Conclusions

This paper has attempted to identify reasons it is so difficult to evaluate the effectiveness of eHealth interventions so that evaluation results are used, relevant, and timely. We note that eHealth interventions themselves, and evaluations of them, have relatively short shelf-lives because of the pace of technological advances, the pace of medical-health care advances, and the production of Internet and electronic resources (eg, apps) by multiple, nontraditional intervention developers, which has increased the number and diversity of intervention types. Such changes may make it difficult to distinguish scientifically validated interventions. Moreover, research on quality and evidence of the effectiveness of eHealth interventions may be anachronistic by the time they are produced and difficult to disseminate to relevant audiences.

This paper has suggested directions that eHealth developers and researchers might take to develop and evaluate eHealth interventions so that their interventions are scientifically grounded, innovative, and attractive enough to be competitive in the eHealth marketplace. This paper also provides guidance for enhancing intervention quality and making information on quality more available to potential users. At this point, these recommendations are designed to spur discussion and further thinking; they are too imprecise and aspirational to constitute a blueprint for change. Also, obstacles stand in the way of their full pursuit and implementation. For instance, some are intrinsically very difficult to achieve (eg, creating highly innovative interventions that anticipate the next wave of technology), and others would be difficult to achieve because they require contributions from multiple stakeholders (eg, creating a consortium of developers, researchers, and others who would grade eHealth intervention quality and promulgate the ratings). However, the first step in overcoming a challenge is to recognize that it exists and then generate ideas to overcome it. The current paper is merely one attempt to encourage progress, especially “quality progress”, in the field eHealth intervention and evaluation.

Continued research on eHealth is critical because such interventions have tremendous potential to provide many patients and families readily available and inexpensive assistance. However, investigators (including ourselves) need to conduct research that recognizes the rapid, ever-changing landscape of technological, scientific, and e-social progress. The notion that technological and scientific progress creates unanticipated change and may render eHealth knowledge and products anachronistic is not new. Unless researchers can discover ways to produce appealing and effective interventions that compete well in the eHealth marketplace, many individuals may use eHealth and mHealth resources that exert negligible or even iatrogenic effects.

## Abbreviations

A-CHESS: Addiction—Comprehensive Health Enhancement Support System
CHESS: Comprehensive Health Enhancement Support System
eHealth: electronic health
e-social: social networking conducted electronically
mHealth: mobile health
RCT: randomized controlled trial
SMART: sequential, multiple assignment, randomized trials
